# The work of local healthcare innovation: a qualitative study of GP-led integrated diabetes care in primary health care

**DOI:** 10.1186/s12913-016-1270-4

**Published:** 2016-01-14

**Authors:** Michele Foster, Letitia Burridge, Maria Donald, Jianzhen Zhang, Claire Jackson

**Affiliations:** 1 School of Nursing, Midwifery and Social Work, The University of Queensland, St Lucia, Qld 4072 Australia; 2 School of Human Services and Social Work, Menzies Health Institute Queensland, Griffith University, Meadowbrook, Qld 4131 Australia; 3 Discipline of General Practice, School of Medicine, The University of Queensland, Level 8, Health Sciences Building, Royal Brisbane & Women’s Hospitals, Herston Road, Herston, Qld 4006 Australia

**Keywords:** Service delivery innovation, Diabetes care, Primary health care research, Qualitative research, Normalization Process Theory

## Abstract

**Background:**

Service delivery innovation is at the heart of efforts to combat the growing burden of chronic disease and escalating healthcare expenditure. Small-scale, locally-led service delivery innovation is a valuable source of learning about the complexities of change and the actions of local change agents. This exploratory qualitative study captures the perspectives of clinicians and managers involved in a general practitioner-led integrated diabetes care innovation.

**Methods:**

Data on these change agents’ perspectives on the local innovation and how it works in the local context were collected through focus groups and semi-structured interviews at two primary health care sites. Transcribed data were analysed thematically. Normalization Process Theory provided a framework to explore perspectives on the individual and collective work involved in putting the innovation into practice in local service delivery contexts.

**Results:**

Twelve primary health care clinicians, hospital-based medical specialists and practice managers participated in the study, which represented the majority involved in the innovation at the two sites. The thematic analysis highlighted three main themes of local innovation work: 1) trusting and embedding new professional relationships; 2) synchronizing services and resources; and 3) reconciling realities of innovation work. As a whole, the findings show that while locally-led service delivery innovation is designed to respond to local problems, convincing others to trust change and managing the boundary tensions is core to local work, particularly when it challenges taken-for-granted practices and relationships. Despite this, the findings also show that local innovators can and do act in both discretionary and creative ways to progress the innovation.

**Conclusions:**

The use of Normalization Process Theory uncovered some critical professional, organizational and structural factors early in the progression of the innovation. The key to local service delivery innovation lies in building coalitions of trust at the point of service delivery and persuading organizational and institutional mindsets to consider the opportunities of locally-led innovation.

**Electronic supplementary material:**

The online version of this article (doi:10.1186/s12913-016-1270-4) contains supplementary material, which is available to authorized users.

## Background

There is a global push for healthcare innovations to address the burden of chronic disease, rising health care costs and problems of quality and health outcomes. Characteristically, healthcare innovations relate to emergent health technologies, new products and services, and altered processes of service delivery [1–3]. The reality of increasing disease burden and healthcare costs is further pressure to implement innovations in service delivery that are also transformative. Greenhalgh and colleagues [4] (p.582) describe such innovations in service delivery as:

“a novel set of behaviors, routines, and ways of working that are directed at improving health outcomes, administrative efficiency, cost effectiveness, or users’ experience and that are implemented by planned and coordinated actions”

Service delivery innovations emerge through different approaches. Top-down policy-driven approaches aim for large-scale system-wide change and are often accompanied by changes to funding arrangements or other financial or performance incentives. For example, the Australian government introduced Medicare items to encourage general practitioners (GP)s to develop coordinated care plans and team care arrangements for patients with chronic disease [5, 6]. Likewise, national policy reforms in the UK over the past two decades have aimed to improve management of complex conditions by specifying the service delivery models to be delivered by health professionals, including GPs [7]. Top-down approaches assume an ordered plan of translation from policy to practice. As such, the translation of top-down solutions into local delivery contexts can yield mixed results [7–11]. Among the many explanations is the unpredictability of how local agents of change will interact, react and organize [12, 13]. Importantly, service delivery innovation also occurs through local, professionally-led or bottom-up approaches [12, 14]. In contrast to top-down approaches, these typically evolve through a more non-linear and disordered, messy process [3, 4, 12], and progress incrementally as adaptability in the local context is key to success [12]. Bottom-up service delivery innovation in professional organizations such as primary care is complicated by the reliance on front-line change agents working collectively rather than individually [10] and organizational processes to support this collective work [15]. Arguably, this form of innovation requires a good understanding of the complexities and nuances of local service delivery innovation as local ‘change work’ which evolves incrementally in unique practice settings [12].

Service delivery innovations that integrate specialist care with primary health care services are increasingly preferred to deliver quality and cost effective care for patients with chronic disease [16]. Likewise, the Australian National Health and Hospital Reform Commission [17] (p.104) recommended “the best models of care for patients that bring together specialists and primary health care professionals to improve health for the most complex patients across all settings, hospitals and the community”. One option is to equip GPs to provide extended clinical services that would otherwise be delivered in the secondary care context [16]. This form of integrated care incorporating GPs with a special interest, has been evident for some time in many areas of primary health care [18, 19] and increasingly in response to the challenges of chronic disease [20, 21], including diabetes [22]. In contrast to traditional disciplinary or sector based ways of working, integrated care typically requires closer collaborations between primary care and specialist services [21, 23, 24] and medical and allied health professionals [25–28]. A key benefit is provision of an alternative source of referral and specialist care in the patient’s local setting [16, 18]. Still, the evidence base is slowly emerging and to a large extent, the challenges surrounding the progression of locally-led service delivery innovations are unknown.

From the evidence established to date, service delivery innovations integrating specialist services in primary care are highly dependent on the commitment of clinicians and their extent of collaboration [14, 29]. Unsurprisingly, service delivery innovations that realign professional boundaries and responsibilities to address system challenges receive varied reactions from health professionals [30, 31]. Martin and colleagues for example [31] have shown how service innovations that attempt to shift the boundaries between specialist and generalist care can threaten taken-for-granted divisions of knowledge and professional roles. While professionals’ appreciation of the need for change in the local context is highly influential [32], their emotional and behavioural engagement with change over time is critical to the outcome [33]. Local champions of change are advocated to drive such change, though their influence is often limited to discrete practice settings unless there is broader organizational support [9]. Indeed, local change is difficult to achieve without appropriate organizational and management support [12, 29, 34]. As previously shown, consensus about organizational priorities [35] and appropriate change incentives [34] accentuate what is valued but more importantly, can galvanize divergent interests. More needs to be known about how innovators operate and interact to influence the progression of locally-led innovations [4] and more so, how their local change work intersects with their unique contexts [2]. To contribute to a stronger evidence base, this research focused on the following question: “What are the perspectives of clinicians and managers involved in local GP-led service delivery innovation for integrated diabetes care, regarding the work of change?”

A GP-led integrated diabetes care innovation was developed in Queensland, Australia to provide diabetes care to patients with complex, unstable diabetes who would otherwise visit a consultant-led hospital outpatient clinic for their diabetes management. Unacceptably long waiting lists for hospital clinics and the belief that with adequate training and support, GPs could provide the required high quality care for people with diabetes underpin the model of care. In this GP-led model, complex diabetes care is provided in the community by a co-located multidisciplinary team comprising an endocrinologist, advanced-skilled GPs (clinical fellows), a credentialed diabetes educator and a podiatrist; with access to other allied health staff on referral depending on patient need. Clinical fellows are experienced local GPs who have undertaken additional postgraduate education in advanced diabetes care. The patient’s referring GP is kept closely informed of all care management and patients are discharged back to them once clinical targets are achieved or after 12 months if it is felt no further improvement can be achieved. The key components and process of care related with this integrated model of diabetes care are fully described elsewhere [36].

In this paper, we report on focus groups with medical and non-medical primary health care professionals and interviews with hospital-based specialists and managers about their perceptions and experiences of a GP-led integrated diabetes care innovation in primary health care. In this case, we were interested in exploring their experiences with an emergent small-scale local innovation to understand the complexities of local innovation work and to inform a broader implementation strategy. The broad aims were to capture multiple perspectives on: a) the meaning and value of the new model of service delivery in the local context; b) the acceptability and compatibility of the new model, with reference to professional relationships, teamwork and organizational capacity; and c) factors likely to impact on local change processes.

## Methods

### Study design and approach

A qualitative research design incorporating focus group meetings and semi-structured interviews was employed to explore the perceptions and experiences of primary health care professionals, hospital-based medical specialists and practice managers involved in a GP-led integrated diabetes care innovation. Using both focus groups and interviews enabled the views of participants in diverse roles to be captured within the data collection timeframe. The focus groups and interviews formed part of a larger mixed method evaluation of integrated primary-secondary care for complex diabetes care in the primary health care setting. Using a randomized control trial [37], eligible patients with complex Type 2 Diabetes are recruited from GP referrals to two metropolitan tertiary hospitals located in Brisbane, Queensland. Three community-based primary care services deliver the GP-led integrated model of care. Usual care is delivered through the diabetes outpatient clinics at the two participating hospitals.

The qualitative study was informed by Normalization Process Theory (NPT). The value of NPT is that it focuses on the meaning that agents of change attribute to new innovations and the work that they do individually and collectively to implement and embed the innovation in day-to-day practice [38]. From this perspective, the work that agents of change do to develop, implement and embed innovations relate to four main analytical concepts: concepts: coherence or sense-making; cognitive participation or engagement; collective action or the work done to enact the innovation; and appraisal of the innovation [38]. Of relevance to this study, the theory provides opportunity to explore and understand the work that local innovators do within unique organizational settings as they progress a service delivery innovation [38]. It therefore assists in uncovering the nuances of change work and the multiple forces shaping the progression of local innovation. Ethics approval was obtained from the Metro Health Service District Human Research Ethics Committee, and the Medical Research Ethics Committee at The University of Queensland. All participants provided informed voluntary consent, and were assured that their anonymity and confidentiality would be protected.

### Participants

Primary health professionals, hospital specialists and practice managers participating in the implementation of the GP-led integrated diabetes care were purposively recruited from two of the three sites where the innovation had been established for at least six months. The third site was established more recently and as such is not included in the analysis. Potential participants were contacted by phone and in writing by one of the researchers (LB) with information about the study and invited to participate in either a focus group or an interview. Twelve participants were recruited across the two sites, including eight primary health care (PHC) clinicians, and two hospital-based endocrinologists and two practice managers (henceforth referred to as stakeholders). Importantly, the sample included all those directly involved with the local innovation in the two sites, with the exception of four PHC clinicians who were unavailable at the time of data collection due to work commitments. In one site, five PHC clinicians participated in focus group discussions and two stakeholders were interviewed (one endocrinologist and one practice manager). In the second site, four clinicians were recruited for the focus group. However, one did not attend on the scheduled day due to work commitments. Two stakeholder interviews were also completed in this site. A description of participants is shown in Table 1.

**Table 1:** Description of participants (*N* = 12)

Characteristic^a^	Stakeholders	Clinicians
	(*N* = 4)	(*N* = 8)
Gender		
Female	2	7
Field (N)		
Medicine	2	4
Nursing	--	2
Dietetics	--	1
Podiatry	--	1
Health administration	2	--
Length of time at this site		
>5 years	2	2
1–5 years	2	3
<12 months	--	3
Hours per week at this site		
<20 h per week	2	4
20–29 h per week	--	1
≥30 h per week	2	3

^a^All participants had trained in Australia

### Data collection and analysis

In each site, focus groups and interviews were conducted approximately six months after commencement of the GP-led integrated diabetes service. To ensure consistency across focus groups and interviews, a topic guide was developed, incorporating key topics and open-ended questions linked to the study aims and conceptual ideas of NPT (Additional file 1). The topic guide included questions to elicit participants’ perceptions and experiences of implementing and working in the integrated care model: experiences of professional, inter-professional and organizational work; the routine activities and resources required to make it work; and perceptions of how it compared to usual care. Opportunity was also provided for participants to make other comments about issues relating to implementation of the innovation. Focus groups were conducted on-site to minimize disruptions for participants. One member of the research team (MF) facilitated all focus groups, with a co-facilitator (LB) to ensure consistency. Each focus group lasted approximately one hour and was transcribed in ‘real time’ by a professional stenographer. Semi-structured interviews with stakeholders were conducted face-to-face by one of the researchers (MF) at a private location chosen by each of the participants. Interviews were on average 30 min in duration and were audio recorded and transcribed in a de-identified form for analysis.

An inductive thematic analysis was conducted on the data set based on the Framework approach [39]. Initially, two researchers (MF and LB) independently read and openly coded the focus group data followed by the interview data. During this process, face-to-face meetings were regularly conducted to discuss and refine codes and agree on a final coding framework, as well as the descriptors of codes. The final coding framework was then applied again across the whole data set by one researcher (LB), noting patterns and contrasts in the data. Commonalities and differences in emergent themes across focus groups and interviews and both sites were discussed as the analysis progressed to elicit an integrated analysis. With reference to the study aims, literature and concepts from NPT, emergent themes were interrogated further to derive how participants made sense of the new model of care and the situated nature of individual and collective activities that surround the work. Exemplar quotes were highlighted during this process.

## Results

From the combined analysis of focus group and interview data, three main themes characterized the perceptions and experiences of the local change work and its value: 1) trusting and embedding new professional relationships; 2) synchronizing services and resources; and 3) reconciling realities of innovation work (Table 2). The first and second themes collectively represent professional, organisational and system-level work that surrounds implementation of local innovations, while the third theme captures the evolving realities of local innovation in an organizational context and the multilevel work required in progressive change. The three themes and exemplar quotes are discussed below. Representative extracts are uniquely coded to distinguish between stakeholders (endocrinologists and managers) and clinicians (clinical fellows, diabetes educators and allied health professionals); for example, S1 indicates stakeholder number 1, and C1 indicates clinician number 1. No major differences were evident in the accounts of participants from the two study sites, though the multi-level analysis revealed different perspectives, which were more indicative of their respective roles and responsibilities.

**Table 2:** Thematic framework

	Theme	Focus
1	Trusting and embedding new professional relationships.	Professional, organisational and system-level work that surrounds implementation of local innovations.
2	Synchronising services and resources.	Professional, organisational and system-level work that surrounds implementation of local innovations.
3	Reconciling realities of innovation work.	The constantly evolving realities of local innovation and the multilevel work required in progressive change.

### Theme1: Trusting and embedding new professional relationships

Across the two sites, most noticeably from the perspectives of PHC clinicians and specialist stakeholders, there was a sense of changing the traditional way of thinking about diabetes care and professional roles as part of innovation work. Managing the traditional specialist-GP divide and the potential divide between the GP-led integrated model and traditional general practice was core work. However, the innovation work was not simply about a service delivery change process. From the experience of one stakeholder there was prolonged work to change the mindset of medical specialists by convincing them about the benefits and quality of delivering complex diabetes care in PHC.

*It’s also overcoming the concept, particularly with tertiary hospital specialists that if it’s in the community, it’s not as complex, and that, therefore, it can't be as good as what we’re doing here. And so changing the specialist concept that actually complicated stuff can happen in the community and that we’re not the ants’ pants and it all happens here… we can manage stuff out in the community… and in fact, potentially even better. And that’s a completely different mindset…[S1].*

The view of this participant was that the reluctance among specialists was in part due to negative experiences with GPs delivering less than expected follow-up care for patients. This possible mistrust was seen as a barrier to address in local innovation work.

*the feeling is that GPs don't know what they’re doing and they’re hopeless and you can't trust them…you ask the GP to do this, this and this, and the patient comes back four months later and nothing has happened. And so, therefore there is this feeling amongst the specialists that the GPs are not doing what they should be doing, and hence we’re the only ones that can do it [S1].*

Yet, there were other views which suggested that the real barriers to building new relationships in diabetes care could be overshadowed by a seeming distrust about competency. For example, entrenched traditional professional roles and referral practices are indicated in the first extract, while the second extract shows a willingness on behalf of specialists to shift from their hospital perspective.

*There are two types of GP, from what I used to see in general practice: High HbA1c they give to the tertiary centre; they do the care. The other type never wants to refer them because they are quite capable. Sometimes they keep it until it is not able to help the patient. The patient's situation is getting worse [C3].*

*That specialist has to be willing to come out of the hospital and see the value in GPs seeing patients in that role [C2].*

Bridging possible divisions and building trust in new professional relationships also extended to GPs not involved in the integrated model. Participants saw the importance of taking steps to improve communication with patients’ regular GPs. To that end, the process of peer-to-peer communication between the model’s clinical fellows and referring GPs was critical.

*Improvement in communication between their regular GP and also breaking down the barriers - a lot of GPs feel intimidated by patients coming through to a specialist clinic and they are loath to change treatment, even though treatment could potentially be changed…In this setting you have got trained GPs talking to GPs so it is breaking down that specialist barrier [C7].*

Likewise, counteracting potential resistance from GPs who could feel intimidated by change was part of the local work. There was a commitment to working steadily with GPs who might feel pressured or under-confident to manage complex diabetes in a different way as the first extract below indicates.

*GPs are feeling a bit threatened, don’t want to deal with all this stuff. We say, ‘actually you can. There are ways of dealing with it’. We take steps to work through like anything [C6].*

This work had the added benefits of earlier access for patients and reduced waiting lists. However, the perception was that this also instilled confidence among specialists for the new model of care, as evidenced by this comment.

*Also for those GPs who are not confident to initiate insulin they always try to get the tertiary centre to see the patient straightaway. There is always a long wait list. We try to reduce the wait list from the tertiary centre so we can see the patient. Upskill the GPs, more confidence from the specialist staff so we can help the patient quicker [C3]*

This theme reveals how the local innovators made sense of the local innovation and their work. This theme also alludes to the potential power of professionals to facilitate or inhibit local innovation [10]. Participants were fully aware that such innovations can be perceived by some as a threat to collective professional identities and traditions [40] and as such, they acted to reinforce the synergies between the model and tradition. Consequently, in working individually and collectively to redefine the scope and boundaries of diabetes care, their work could be interpreted as negotiating and building trust in new kinds of professional relationships.

### Theme 2: Synchronizing services and resources

Participants endorsed the benefits of GP-led integrated diabetes care compared to the delineation between secondary and primary care. The consensus was that the innovation was *a one-stop shop* [C5], with *a mixture of general practice and specialist based diabetes care in a multidisciplinary model* [S3]. The integrated approach provided a way of synchronizing a complex care pathway and fragmented system for the benefits of patients. This comprised multiple elements: *fast and convenient…access* [S3]*; familiar and comfortable* [S1]*; personalized setting* [C7]*; comprehensive approach* [C8]*; continuity of care…and a saving of time* [C3]; and *time with them* [C8]*.* In taking specialist care out to the patient, the integrated model streamlined and routinised care processes making it easier for patients to access care.

*It puts it closer back to where the patient is. It assures them of some continuity so they come here; usually see the same person each time. There is an ease of coming here. They have a relationship with the diabetic educator. They have quicker access so they can have repeat return visits within two or four weeks [C4].*

In this case, the diabetes educator was seen by stakeholders to enhance continuity of care and relationships. For other PHC participants within the team, the diabetes educator was an identifiable *resource person…and font of information* [C6] and *the organizer and doer of the clinic* [C2], and thus, critical to its functioning.

However, participants also acknowledged the collective work involved in harmonizing the new model of service delivery with routine general practice. Good communication and information sharing about patient care was core work. Ensuring a consultation summary *gets sent to the GP immediately* [C7] was also empowerment work, reinforcing the complementarity of integrated care with general practice.

*I would like to think that it is also disseminating information back to the GP and empowering them to be able to continue with the care of these people who many have been complicated at a stage [C6]*

Nevertheless, restrictions on comprehensive sharing of patient information across public and private sectors and secondary and primary care settings were perceived to be system impediments. From all perspectives, this was compounded because patients had multiple issues involving multiple providers, and from the stakeholder perspective, it limited holistic care.

*A lot of [patients with diabetes] have other co-morbidities as well. So actually finding out what their nephrologist and what their cardiologist and some of these other players are doing with the patient is still a frustration, because we don’t necessarily have complete access to the full patient history and full medical record [S2].*

Managing the case mix and patient flow was also necessary collective work to harmonize but also properly differentiate the integrated model from routine general practice. The PHC participants in particular perceived ongoing work to differentiate those patients with complex, unstable diabetes who could benefit from more intensive, time-limited integrated care, with a specific objective.

*It depends on the degree of complexity. If someone arrives and has been well controlled, we start talking about, “your GP is doing a great job” and we send them back to them quite quickly. If it is something all over the shop, we keep seeing them and keep telling the GP to keep an eye on things in between [C6].*

From all perspectives, there was ongoing work to get the necessary resources to make the local innovation operate successfully. This included a skilled workforce, which one participant perceived to be an individual responsibility *to learn something extra* [C4]. Other participants reinforced the collective responsibility to produce a highly skilled workforce.

*You need to have GPs themselves upskilled to some extent, so they do have some additional training in the diabetes area. You need diabetes nurse educators who are capable of working reasonably independently at pretty high-level organisational skills to shuffle people through the clinic [S3]*

However, the realities of the broader environment were also highlighted by one stakeholder concerned with the fit longer-term between the local innovation and the *small business environment.*

*This is hard medicine and you’re going to be paid about a third to a half of what you would earn in another setting. This is not an attractive equation unless you’re particularly motivated about the reasons you get into medicine…so selling what this model is to people who would then be prepared to work in it is tough [S2].*

This theme sheds light on some of the opportunities and constraints inherent at the organizational and broader healthcare system levels and which innovators confront in change processes. While the legitimacy and progression of locally-led innovation may rely on professional leadership and building trust within local networks [15], there is a complex interplay with these broader levels. As an example, in the Australian context where fee-for-service dominates primary health care, incentives that engage others in innovation are an important consideration [6]. However, of particular interest is how front-line innovators manage these dynamics and this is addressed in the third theme.

### Theme 3: Reconciling realities of innovation work

In the case of this innovation, the realities of additional administrative work, staffing limits and time pressures were commonplace for many participants and required flexibility. From the PHC perspective, there was *a lot more administrative work to do* [C7]. This reality and the associated time pressures were highlighted as major differences compared to usual care. Yet, the tasks of collecting and collating complete clinical information, communicating to the team, and to the regular GP, were integral to the authenticity of the model.

*The only problem that mostly comes up is that time management issue of having to try and see them, talk to [other team members]….write a letter back to the GP. Writing the letter I found is quite difficult…You want to be thorough. You are imaging from the GP’s perspective [C1].*

The additional administrative load associated with communication with patients’ regular GPs was a *fairly time-intensive component* [S2]. However, according to this stakeholder, in some instances additional liaison work was generated by poor quality referrals to the integrated model of care.

Though all participants viewed the staffing capacity within the integrated model as a distinct improvement on usual diabetes care, it was evident at both sites that there was a need to balance the ‘ideal’ model with the realities of resourcing. A major issue at both sites was the diabetes educator allocation of time and tasks. According to one participant there were tensions trying to enact the full scope of care within the boundaries of the staffing allocation and expected blend of clinical and administrative responsibilities.

*I think splitting the admin role from the education role might be beneficial…Because it limits the educator's time to be able to do the primary role, in other words, education, some administrative appointment chasing up and that sort of thing organised by someone who is not the clinical person might be more efficient [C6].*

Although the integrated model was compared favourably to usual care, where it was perceived that patients *don’t get a lot of contact with the diabetes educator* [C7], some perceived the less than optimal staff time generating discretionary work. For example, one participant recounted being *selective with the patients that I think need a bit extra time* [C7]. Furthermore, in view of one stakeholder, the time allocated for the diabetes educator was less than optimal to address the extent of follow-up care inherent in the local integrated care model.

*I think in our setting the time allocation that the diabetes educator has been a bit light on, it’s…been half time which essentially means two days one week, three days the next. And probably three days per week would be a more reasonable thing. It’s just because there is a lot of follow-up, there’s a lot of ambulatory, stabilization of insulin, that sort of thing, which requires time and can’t be done on clinic days [S3].*

Likewise, it could be difficult to manage the complexity of patient issues when *there is a limitation on the expectation of time [spent] on the initial consultation [C7]*. These examples reveal the organizational and health system parameters in which micro-level innovation work takes place and which clinicians must negotiate on a daily basis. In these instances the discretionary practices around patient time and activities are revealed.

Aside from these contextual issues, patient attendance was an ongoing reality for participants to manage. In these locally-led service delivery innovations, where patients could be confused about the new model and various professional roles, *a model of engagement with patients* [C3] and efforts *to try and engage patients locally* [C1] were essential innovation work. This involved constant education about roles, standardised follow-up and reminders, but more so, flexibility. It was time-consuming and required resource coordination, but was essential to change the mind set of patients.

*The clinic patients, there is a mind-set that we are trying to train…Every week it will happen that I ring and ring and ring and they don't answer and eventually they decide to…It has been a big coordinating role. The [diabetes educator] position….is obviously part of that role. All of that takes you away from face-to-face with the patient [C7].*

Yet, the collective work was more than simply changing how patients understood and engaged with their diabetes care. Unquestionably, it was also about convincing them of the value of the integrated model compared to usual care to *keep up the patient numbers* [S4]. Across all perspectives, this was fundamental to the financial viability of the model.

*I felt pressure for us to be a financial model. It has to be economical for the practice. In many ways it is not…sometimes patients don’t turn up…at times it is a lot of juggling. It takes a lot of time. We have to move patients to a clinic to fill it up to warrant the staff or we cancel the clinics…if you keep cancelling the patient it becomes dangerous to them. That is what has to be considered for this model [C7].*

Equally, participants across both sites recognized the work in changing a system mind set. For some PHC and stakeholder participants there was a sense of individual responsibility to drive change in the local context. For one participant, the locally-led integrated care innovation was difficult work because of minimal interest, and consequently, this elevated individual responsibility for generating ideas about new ways of service delivery and bringing consensus in the local context.

*…there are fewer of us, and probably me largely, who are really driving systematisation innovation change. There are a few of us interested in this space. We will come up with ideas and present them to the group, not necessarily formalised. We work our way around everybody [C4)].*

Similar to bringing local people around, there was an appreciation of the work required to gain consensus across all disciplines to ensure everyone is *aiming for the same outcome* [S4]. The PHC participants and specialist stakeholders also reinforced the necessity of local leadership to engage and convince the various stakeholders across all levels of the system. One participant clearly brought to light the challenge of changing an organizational and institutional mindset historically framed on the values of autonomy and tradition.

*You have clinicians, clinician engagement and nursing engagement in a general practice. The key thing will be engaging of the clinician. Because general practice is very autonomous you need to have everyone onside and behind it. Change is hard. People are resistant. Medicine is particularly resistant to change. Getting them to hand over certain things, change behaviours, submit to audit, all that sort of stuff is challenging. If you can get everyone engaged in that process then it is very exciting, success breeds success [C4]*

This theme accentuates the realities of emergent tensions in innovation work across micro-level, organizational and healthcare system boundaries. It is insightful about the pivotal roles that front-line clinicians occupy in managing these boundaries. The critical roles of change champions and boundary navigators are evident, as is the weakness of locally-led innovation if these people are lacking. Consequently, this theme also uncovers the potential risks to the progression and legitimacy of locally-led service delivery innovations.

## Discussion

This exploratory study on a GP-led integrated diabetes care innovation in primary health care captured the perceptions and experiences of primary health professionals, hospital specialists and practice managers. The findings as a whole provide further insight into the professional and organizational aspects of local change work [41]. More so, the findings reinforce the interplay of micro-level change practices and contextual, or macro-level, elements as a local innovation evolves and takes shape within the local delivery context [2, 4]. In relation to the empirical evidence base on locally-led integrated care innovations, the findings evoke three main considerations. The first theme, trusting and embedding new relationships, is a reminder that while locally-led innovation is designed to address local problems, convincing others of its value is core work. This is particularly so when the innovation challenges professional norms and involves changes to traditional delivery models and renegotiation of professional roles [30]. In this case, the findings are consistent with previous research which has indicated that the success of such innovations is dependent on the trust of all involved and the credibility of clinicians [20]. Previous research has also shown that clinical and organizational champions can play a pivotal, albeit limited, role in this regard. This can be seen, for example, in promoting and leading local innovation [42] and motivating diverse interests within the local context [9]. Likewise, in the current study this was reinforced by one stakeholder. Yet, the findings also imply a much deeper, potentially resistant mindset, consistent with research by Ferlie and colleagues [10], which highlighted the influence of macro institutional processes on micro-level professional identities and work practices. This suggests that the progress and success of the GP-led diabetes care innovation in local contexts will requires strategies at both the macro and micro-levels to enhance interprofessional learning, build trust and bridge professional divides.

A further consideration relates to the importance of organizational and health system factors to the evolution and progression of a professionally-led local innovation, confirmed in the second and third themes. Unlike some innovations that are highly contained to micro-level changes in professional practice [10], this GP-led diabetes care innovation required a complex set of changes at the organizational and system levels. At the organizational level, issues relating to increased workloads, referral relationships, time constraints and financial matters resonate with the research to date which has highlighted the determining influence of contextual factors [27, 43]. Indeed, the findings as a whole indicate that while internal processes can drive local innovation to a degree, these processes are not immune from external realities, rather there is an “interaction between external/macro and internal micro developments” ([12], p.210). Furthermore, as is evident in the current findings, staffing resources can impact innovation work and more so, generate judicious decisions about prioritization of tasks and allocation of time. While adaptation is to be expected as the innovation evolves, it is not known how discretionary management of inherent tensions impacts on the quality of patient care or the fidelity of the innovation.

Importantly, as the findings suggest, this bottom-up locally-led service delivery innovation represents a novel process innovation [3] in this case in diabetes care, in that it seeks to value-add by way of improving efficiency and timely access and not supersede other specialist and generalist care components. Nonetheless, innovations in one part could be threatening to another part of the system [3]. The references to clinicians’ hesitancies about changes to the delivery model may in part be due to this factor. Similar to the recommendation of a recent study on healthcare service change [44], the acceptance and indeed continued evolution of this locally-led innovation will depend on demonstrating its value to the broader healthcare community. This requires evaluation research but more so, a capacity within the local innovation to use research persuasively to promote what starts out as an answer to a local problem recognition [3].

This study provided an opportunity to rigorously evaluate an emergent bottom-up service delivery innovation, drawing on clinicians’ and managers’ perspectives. However, the findings must be interpreted within the limitations of the study. In this case, the study involved two sites and 12 participants, though this included the majority of those directly involved in ongoing small-scale local innovation work. Although the findings provide valuable insights into an emergent small-scale local innovation, the findings are not transferable to other settings. On the other hand, the application of NPT allowed an exploration of the nuances of individual and collective work to progress the innovation, from multiple perspectives across clinical and organizational levels. Though this is limited to a small-scale innovation within specific contexts, nonetheless, the theory is valuable in eliciting the dynamic conditions and dimensions of innovation work for focus in formal evaluation [38]. Future research incorporating a multi-level longitudinal perspective would provide an opportunity to examine the progression and spread of the innovation and more so, the evolution of engagement, relationships, roles and resources around this local service delivery innovation.

## Conclusions

This study contributes much needed knowledge about the complexities of locally-led service delivery innovation. Although the micro-level innovation work is often complicated by organizational and broader systemic factors, nonetheless, innovators can and do act internally and externally, and proactively, to progress the innovation. However, the key lies in building consensus and coalitions of trust at the point of service delivery and longer-term persuading organizational and institutional mindsets to consider the opportunities of locally-led service delivery innovation.

## Additional file

Additional file 1:
**Interview Guide.** (DOCX 19.5 kb)

## Abbreviations

GP: General Practitioner
NPT: Normalization Process Theory
PHC: Primary health care
