# Supplementary material for: The work of local healthcare innovation: a qualitative study of GP-led integrated diabetes care in primary health care
Source: BMC Health Serv Res. 2016 Jan 14;16:11. doi: 10.1186/s12913-016-1270-4 (PMC4712472; doi:10.1186/s12913-016-1270-4)
Supplement: Additional file 1: — Interview Guide. (DOCX 19.5 kb) [file 12913_2016_1270_MOESM1_ESM.doc]

**INTERVIEW GUIDE: Health Professionals**

**General Awareness/Early impressions**

Thinking generally about the test model:

- What do you understand as the purpose and benefits of the test model?
- What are the main components of the ‘test model’ in this setting?
- What is distinctive about the test-model and how is it different to other types of diabetes care in primary care settings?

**Compatibility and acceptability of model as a clinician**

Thinking now as a clinician about the compatibility of the test model with usual care for type 2 diabetes patients

- How does it compare with usual care: what is required of you as clinicians? E.g. tasks, knowledge, skills, time, responsibilities
- Can you tell me whether you did anything additional/specific to meet these requirements?
- Tell me about the fit (of test model) with your day-day practice?
- As a clinician, what aspects of the test model are you concerned about?

**Compatibility/acceptability relational aspects of the test model**

- How does the diabetes team actually work together currently?
- What influence does the test model have on your teamwork?
  - E.g. relationships; communication; decision-making; team learning?
- What aspects of the test model are you concerned about for teamwork?

**Compatibility and acceptability of test model from organisational aspect**

Thinking now about this practice setting/this organisation

- How do you see the ‘test model’ affecting the roles, responsibilities, relationships within the local practice setting? And those beyond your practice?
- How does it fit with the overall activity/goals of this practice?
- What aspects of the test-model are you concerned about for this practice? E.g. resources; Cost to patient? Access? Long-term follow-up?

**Processes and factors likely to enhance or inhibit adoption and sustainability**

**Your experiences in implementing evidence-based clinical team care.**

- As individuals and as a group, how do you keep up to date with changes in diabetes care?
- How do you go about sharing new knowledge with one another as a team?

**Your experiences with organisational change**

- What things make it easier for that change to take place?
- What makes it difficult to change current practices?
- What are the key attributes of successful and sustainable change processes?

**Closing**

Is there anything you would like to add that we have not covered? Thank you
